# DNA-methylation subgroups carry no prognostic significance in ATRT-SHH patients in clinical trial cohorts

**DOI:** 10.1007/s00401-023-02614-9

**Published:** 2023-07-31

**Authors:** Quynh T. Tran, Santhosh A. Upadhyaya, Catherine A. Billups, Arzu Onar-Thomas, Md. Zahangir Alom, Steven S. Carey, Giles W. Robinson, David W. Ellison, Amar Gajjar, Brent A. Orr

**Affiliations:** 1 Department of Pathology, St. Jude Children’s Research Hospital, 262 Danny Thomas Place, MS 250, Memphis, TN 38105 USA; 2 Department of Pediatrics and Communicable Diseases, C.S. Mott Children’s Hospital, University of Michigan, Ann Arbor, MI 48109 USA; 3 Department of Biostatistics, St. Jude Children’s Research Hospital, Memphis, TN 38105 USA; 4 Department of Hospitalist Medicine, St. Jude Children’s Research Hospital, Memphis, TN 38105 USA; 5 Department of Oncology, St. Jude Children’s Research Hospital, Memphis, TN 38105 USA

Atypical teratoid/rhabdoid tumors (ATRT) are malignant embryonal tumors, most often presenting in children under three years of age [7]. Three consensus ATRT molecular subtypes have been established, designated ATRT-SHH, ATRT-MYC, and ATRT-TYR [4, 5]. The ATRT-TYR group has been associated with better overall survival [3, 10]; whereas, patients with ATRT-SHH have inferior outcomes, largely attributable to an increased risk of metastasis [10]. Recently, Federico et al. analyzed a registry cohort of ATRT-SHH and reported three molecular subgroups designated ATRT-SHH 1A, 1B, and 2 [2]. While some novel molecular classes may represent important determinants of clinical outcome or surrogates of alternative driver mutations [6], others may reflect biologically driven differences, but lack clear clinical import. To validate the three ATRT-SHH molecular subgroups and add insight into the clinical significance of these molecular classes, we evaluated the clinical features and outcome data for ATRT-SHH patients treated in St. Jude Children's Research Hospital (SJCRH) initiated multi-institutional clinical trials (*n* = 39): SJYC07, SJMB03, SJATRT, or in a consortium trial open at St. Jude: PBTC-001 (*n* = 2). Patients equivalently treated on non-protocol treatment plan (NPTP) (*n* = 2) at St. Jude were also included where available.

First, we validated three subgroups of ATRT-SHH using publicly available datasets containing ATRT-SHH tumors [1, 3, 4] (Supplemental Methods and Fig. S1). Next, we assigned consensus labels to SJCRH cases using a combination of unsupervised and semi-supervised methods [8–10] (Fig. S2). Consensus subgroup labels were reliably assigned to 41/43 cases, with 14 designated ATRT-SHH-1A, 11 ATRT-SHH-1B, and 16 ATRT-SHH-2 (Table S1). Analysis of the clinical data from these 41 patients supported the previous observations of subgroup-specific differences in tumor site (*p* = 0.005) [2]. We observed an enrichment of ATRT-SHH-2 in the infratentorial compartment and ATRT-SHH-1 in the supratentorial compartment (Table S1). We also confirmed the previously observed difference in age at diagnosis with the ATRT-SHH-1B patients presenting at a median age of 3.5 years, compared to 1.1 years for both ATRT-SHH-1A and ATRT-SHH-2 (*p* = 0.008). No differences were identified with respect to gender or germline cancer predisposition among subgroups (Table S1).

Next, we evaluated the patient outcomes for the thirty-six patients who enrolled on frontline trials or received NPTP equivalent to frontline protocols at diagnosis, including SJYC07 (*n* = 27), SJMB03 (*n* = 5), and PBTC-001 (*n* = 4). In contrast to the findings reported by Federico and colleagues [2], we did not observe differences in survival, including overall survival (OS) or progression-free survival (PFS) (Fig. 1a, b). We also evaluated ATRT-SHH subgroups for survival differences after considering metastatic status at presentation or with respect to post-recurrence survival but found no significant difference (data no shown).

**Fig. 1:**
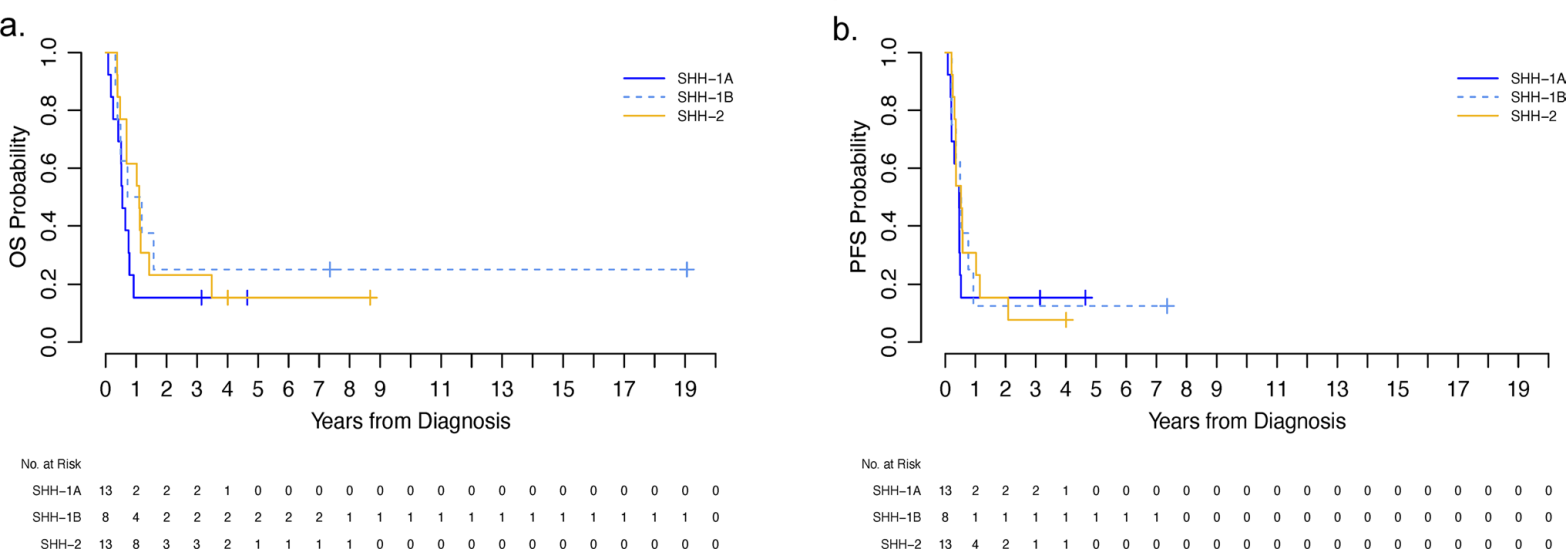
Kaplan–Meier survival curves for combined frontline trial patients. Five-year OS (**a**), PFS (**b**) by ATRT-SHH molecular subgroup

In summary, our findings validated reported substructure within the ATRT-SHH molecular group and confirmed the primary reported clinical correlates of enrichment for infratentorial location in the ATRT-SHH-2 subgroup and an older age of presentation in the ATRT-SHH-1B subgroup [2]. However, we found no significant difference in patient survival among subgroups in frontline clinical trials. There are several possible explanations for the differences observed between our cohort and the previously published cohort. First, our cohort contains significantly fewer patients and, therefore, may be underpowered to detect group-specific survival signal. Additionally, differences in therapeutic interventions could be masking subgroup-specific outcome differences. The ATRT-SHH-1B patients previous report had a significantly older median age compared to our cohort (8.9 years old compared to 3.5 years, respectively), increasing the proportion of cases eligible for craniospinal irradiation (CSI). We previously reported that ATRT patients receiving upfront CSI show significantly better survival compared to patients receiving focal postoperative radiation alone [10]. Although we find no support in our cohorts that the ATRT-SHH subgroups should be used as the basis for trial stratification or clinical decision-making, it will be important to monitor for clinical impact in subsequent studies.

## Supplementary Information

Below is the link to the electronic supplementary material.

Supplementary file1 (PDF 402 kb)

## Data Availability

The data that support the findings of this study are available from the corresponding author (B.A.O) upon reasonable request.
