# Supplementary material for: DNA-methylation subgroups carry no prognostic significance in ATRT-SHH patients in clinical trial cohorts
Source: Acta Neuropathol. 2023 Jul 31;146(3):543–5. doi: 10.1007/s00401-023-02614-9 (PMC10412479; doi:10.1007/s00401-023-02614-9)
Supplement: Supplementary file 1 — Supplementary file1 (PDF 402 kb) [file 401_2023_2614_MOESM1_ESM.pdf]

Supplementary Methods

Processing methylation data

Raw IDAT files from different studies (GSE90496 [1], GSE141039 [2], GSE141363 [3], and GSE70460 [4]) were downloaded from the Gene Expression Omnibus database (https://www.ncbi.nlm.nih.gov/geo/). After removing duplicates, 256 unique IDAT files were used for further analysis. Methylome from 47 SJCRH samples were collected using the human Infinium MethylationEPIC BeadChip array (Illumina, San Diego, CA) on 250-500 ng of DNA extracted from formalin-fixed paraffin-embedded (FFPE) tissues. Samples were handled in accordance with the Illumina Infinium HD Methylation Assay Protocol, as previously published [8].

All methylation data were processed in R (http://www.r-project.org, version 4.0.2), using several packages from Bioconductor and other repositories. Specifically, all arrays were preprocessed using the *minfi* R package (v1.36.0). Background correction with dye-bias normalization was performed for all samples using noob (normal-exponential out-of-band) with a “single” dye method with preprocessFunNorm. Probe filtering was performed after normalization. Specifically, probes located on sex chromosomes containing a nucleotide polymorphism (dbSNP132 Common) within five base pairs of and including the targeted CpG-site or mapping to multiple sites on hg19 (allowing for one mismatch), and cross-reactive probes were removed from the analysis. After the filtering process, 438370 probes remained.

Methylation classification using unsupervised and semi-supervised models

We first performed dimensionality reduction and visualization using *t*-distribution stochastic neighboring embedding (*t*-SNE) analysis on the 256 publicly available methylation data in order to validate the methylation classes within the ATRT family (i.e. TYR, MYC, and SHH) (Supplemental Fig. 1). The distance matrix of 1 - Pearson’s correlation of 5000 variably methylated probes were used for *t*-SNE analysis with these parameter settings: dims=20, perplexity=16.5, and theta=0.6. Among the 256 ATRT samples, 97 samples were identified by previous studies to be in the SHH group. These 97 samples were combined with the 47 SHH samples from the SJCRH for classification analysis.

To determine the subgroups for the SHH samples, we performed an unsupervised analysis using the *cola* R package (v.2.2.0) and a semi-supervised analysis using the *ssc* R package (v.2.1.0). All 144 SHH samples were clustered using the *run_all_consensus_partition_methods* function

with partition_method= c("skmeans", "NMF", "kmeans"), top_value_method = "SD", top_n= c(5000, 10000, 20000). Semi-supervised models have been shown to effectively label or relabel methylation samples utilizing public methylation data. There were 31 publicly available samples with unknown SHH subgroups or their labels were not in agreement with the clustering results. These 31 samples were used as a transductive set in the semi-supervised analysis. The 47 SJRCH samples were used as an inductive test set. In this study, we trained 4 different semi-supervised models (selfTrainning_oneNN, setred_SVM, snnrce, and triTraining_oneNN) [5] on genome-wide DNA methylation profiles of the 97 SHH samples (Supplemental Figure 2). Labels for the 31 unknown SHH samples and the 47 SJRCH samples were determined when there was a consensus from at least 4 out of 7 unsupervised and semi-supervised models.

Statistical Methods

Chi square tests were used to examine associations among categorical variables. Wilcoxon rank sum tests and Kruskal-Wallis tests were used to examine the association between age by protocol and SHH subtype. For frontline protocols, survival was defined as the time interval from diagnosis to death from any cause or to date of last contact for survivors. Progression-free survival (PFS) was defined as the time interval from diagnosis to date of first relapse or progression or to the date of death from any cause, or to the date of last contact for patients without disease progression or death related to disease. Event-free survival (EFS) was defined as the time interval from diagnosis to date of first event, where an event included disease progression or relapse, second malignancy, secondary tumor, or death from any cause, or to the date of last contact for patients without events. For patients with recurrent disease and enrolled on SJATRT, the start of the OS and PFS interval was date of treatment start on SJATRT. Outcome distributions were estimated using the method of Kaplan and Meier and standard errors were calculated using the method of Peto and Pike. Differences in outcome distributions were examined using log rank tests.

Table S1. Patient characteristics for all patients (frontline and recurrent) by SHH subtype

	SHH Subtype						All Patients		*p*
	SHH-1A		SHH-1B		SHH-2				
	n	%	n	%	n	%	n	%	
Study									
PBTC-001/NPTP	1	7.1	2	18.2	1	6.3	4	9.8	--
SJMB03	1	7.1	3	27.3	0	0	4	9.8	
SJYC07	11	78.6	3	27.3	12	75.0	26	63.4	
SJATRT	1	7.1	3	27.3	3	18.8	7	17.1	
Sex									
Female	5	35.7	6	54.5	11	68.8	22	53.7	0.19
Male	9	64.3	5	45.5	5	31.3	19	46.3	
Site Group									
Infratentorial	3	21.4	0	0	9	56.3	12	29.3	0.005
Supratentorial	11	78.6	11	100.0	7	43.8	29	70.7	
Germline Alteration									
Positive	4	36.4	3	37.5	6	54.5	13	43.3	0.64 (+ vs. -)
Negative	7	63.6	5	62.5	5	45.5	17	56.7	
NA	3	--	3	--	5	--	11		
Total	14	100.0	11	100.0	16	100.0	41	100.0	

Supplementary Figures

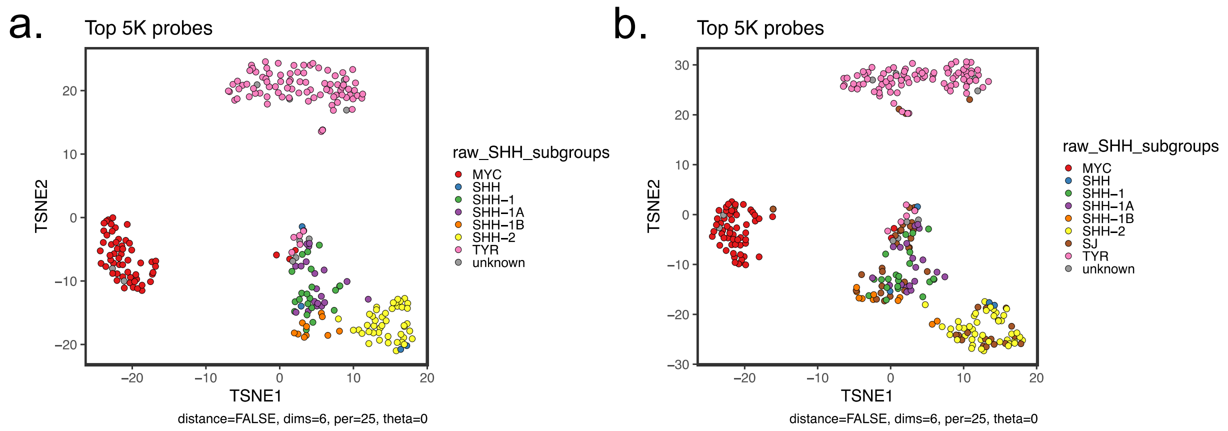

Figure S1. *t*-distribution stochastic neighboring embedding (*t*-SNE) visualization (a) of 256 ATRT samples from the GEO database (b) with SJ samples projected on 256 GEO samples. The distance matrix of 1 - Pearson's correlation of 5000 variably methylated probes were used for *t*-SNE analysis with these parameter settings: dims=20, perplexity=16.5, and theta=0.6.

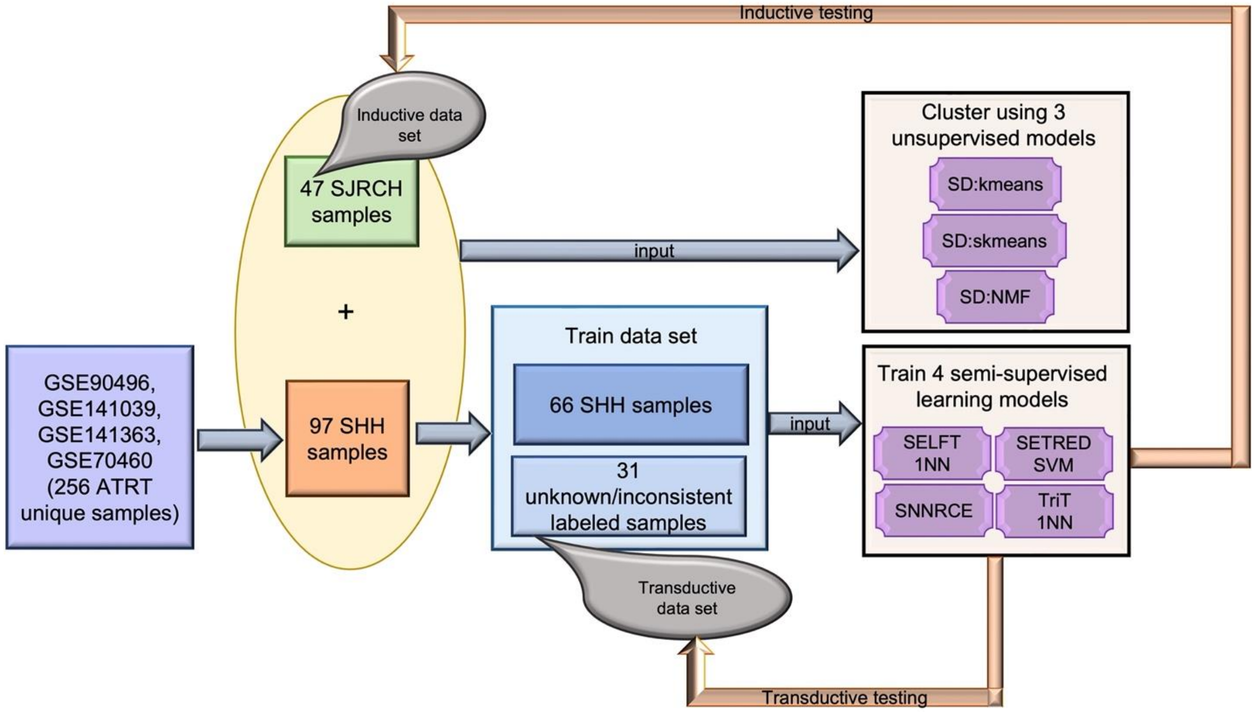

Figure S2. Process of determining SHH subgroups. Raw methylation data from GSE90496, GSE141039, GSE141363, and GSE70460 were downloaded from the Gene Expression Omnibus

database (https://www.ncbi.nlm.nih.gov/geo/). There were 256 unique IDAT files were used for further analysis. Among the 256 ATRT samples, 97 samples were identified by previous studies to be in the SHH group. These samples were combined with the 47 SHH samples from the SJCRH for classification analysis. The combined 144 samples were clustered using 3 different unsupervised models (kmeans, skmeans, and NMF). We trained 4 semi-supervised models (SELFT_1NN, SETRED_SVM, SNNRCE, and TriTrain_1NN) using 97 publicly available samples. The trained models were then used to predict the labels of the 47 SJCRH samples (the inductive data set).
